# Exploring the parasite load and molecular diversity of *Trypanosoma cruzi* in patients with chronic Chagas disease from different regions of Brazil

**DOI:** 10.1371/journal.pntd.0006939

**Published:** 2018-11-12

**Authors:** Ícaro Rodrigues-dos-Santos, Myllena F. Melo, Liane de Castro, Alejandro Marcel Hasslocher-Moreno, Pedro Emmanuel A. A. do Brasil, Andréa Silvestre de Sousa, Constança Britto, Otacilio C. Moreira

**Affiliations:** 1 Laboratório de Biologia Molecular e Doenças Endêmicas, IOC /Fiocruz, Rio de Janeiro, Brazil; 2 Laboratório de Pesquisa Clínica em Doença de Chagas, INI/ Fiocruz, Rio de Janeiro, Brazil; Sacro Cuore Hospital, ITALY

## Abstract

Chagas disease is still a major public health issue in many Latin American countries. One of the current major challenges is to find an association between *Trypanosoma cruzi* discrete typing units (DTUs) and clinical manifestations of the disease. In this study, we used a multilocus conventional PCR and quantitative real time PCR (qPCR) approaches to perform the molecular typing and parasite load quantification directly from blood specimens of 65 chronic Chagas disease patients. All patients were recruited at the same health center, but their place of birth were widely distributed in different geographic regions of Brazil. Of the 65 patients, 35 (53.8%) presented positive amplification by real time qPCR, being 20 (30.7%) with the clinical indeterminate form and 15 (23.1%) with the cardiac form of the disease. The parasite load median for all positive patients was 2.54 [1.43–11.14] parasite equivalents/mL (par. Eq./mL), with the load ranging from 0.12 to 153.66 par. Eq./mL. Noteworthy, the parasite load was significantly higher in patients over 70 years old (median 20.05 [18.29–86.86] par. Eq./mL). Using guanidine-EDTA blood samples spiked with reference *T*. *cruzi* strains, belonging to the six DTUs, it was possible to genotype the parasite up to 0.5 par. Eq./mL, with high specificity. Of the patients with positive qPCR, it was possible to identify the *T*. *cruzi* DTU in 28 patients (80%). For the remaining patients (20%), at least a partial result was obtained. Analysis of specimens showed prevalences of TcVI, TcII and mixed infection TcVI+TcII equal to 40%, 17.1% and 14.3%, respectively. In addition, two patients were infected by TcV, and one patient was coinfected by TcIII+TcVI, These last three patients were in stage A of chronic chagasic cardiomyopathy (CCC), and they were born at the Bahia State (northeast region of Brazil). When *T*. *cruzi* genotypes were compared with the parasite load, more elevated parasite loads were observed in patients infected by TcII in general (parasite load median of 7.56 par. Eq./mL) in comparison to patients infected by TcVI (median of 2.35 par. Eq./mL). However, while the frequency of CCC was 50% in patients infected by TcVI and TcV, only 16.7% of patients infected by TcII evolved to CCC. Taking together, our results contribute to update the epidemiological knowledge of *T*. *cruzi* DTUs in Brazil, and highlight the age of patient and infection by TcII as important features that lead to the observation of higher parasitemia levels.

## Author summary

Chagas disease is a neglected illness caused by the protozoan parasite *Trypanosoma cruzi*, which affects millions of people in the endemic countries in Latin America. Despite the knowledge about the parasite genetic diversity, there still some gaps regarding the association between those different genotypes and clinical manifestations, including the level of parasites circulating in the bloodstream. From a group of adult chronic patients presenting the cardiac and indeterminate forms, originated from different regions of Brazil, we evaluate the parasite load and *T*. *cruzi* genotyping in order to search an association with the clinical features of Chagas disease. We observe a high diversity of *T*. *cruzi* genotypes in Brazil, although the genotypes TcVI, TcII and mixed infection TcVI+TcII were more prevalent, but no correlation with the cardiac or indeterminate clinical forms was observed. In contrast, we found that the age of patient and infection by TcII are associated to higher parasite load levels.

## Introduction

Chagas disease (CD) is considered the most important parasitic infection in Latin America, with serious consequences for public health and national economies [1]. It is caused by the flagellate protozoan *Trypanosoma cruzi* and mostly affects poor populations in 21 countries from the endemic areas. It is estimated around 6 million to 7 million people infected worldwide and 75 to 90 million exposed to infection by the parasite [2, 3]. In the chronic phase, the disease shows a diversity in clinical manifestations, from indeterminate to cardiac and/or digestive forms [2, 4], which can be associated to complex interactions between the genetic diversity of the parasite and the host and environmental and epidemiologic factors [5]. Nevertheless, biomarkers for disease progression and prognosis are still unavailable to patients directly [6].

*T*. *cruzi* is represented by a set of populations showing different levels of pathogenicity, sensitivity to drugs and disease prognosis [7–11], as well as eco-epidemiological complexity [12, 13, 14]. In 2009, the scientific community achieved a consensus to classify *T*. *cruzi* isolates into six discrete typing units (DTUs)—*T*. *cruzi* I to VI—based on different molecular markers and biological characteristics of the parasite [15].

So far, TcI has been associated with the cardiac form of the disease in Argentina, Colombia, Brazil and Venezuela [16, 17, 18] while TcII, TcV and TcVI were reported to all clinical manifestations. TcIII and TcIV are commonly associated to sylvatic cycles, being related to oral outbreaks at the Amazon region and Venezuela [19, 20, 21]. Nevertheless, Martins et al. [22] reported three patients with the chronic indeterminate form at Rio Grande do Norte State (Northeast region of Brazil) who were infected by TcIII.

Quantitative PCR is generally used to monitor the parasite load during etiological treatment and could be particularly valuable as an indicator of therapeutic failure in Chagas disease [23, 24]. The current real time PCR assays have shown satisfactory sensitivity and accuracy for the detection and quantification of *T*. *cruzi* from peripheral blood samples of infected patients, even in the chronic phase of the disease [25, 26, 27]. Recently, the analytical validation of a consensus real time PCR assay for the quantification of parasite load from blood samples of Chagas disease patients was published [28]. In that study, chronic patients from different countries and infected with distinct parasite genotypes were evaluated. Using *T*. *cruzi* satellite DNA or kDNA as target, no significant difference was observed in the parasite load median between symptomatic and asymptomatic patients, or for patients infected with distinct DTUs, even observing a wide parasitemia range among the chronic patients. It was in agreement with previous observations that showed no correlation between *T*. *cruzi* parasitemia and the clinical form of the disease [29, 30, 31]. However, this wide range of parasite load and its association with the *T*. *cruzi* genotyping and severity level of the disease in patients with CCC from different localities in Brazil were no further explored yet.

Herein, the parasite load and molecular diversity of *T*. *cruzi* were directly assessed from blood samples of patients born in different states from Brazil, presenting different levels of CCC, who were being assisted at outpatient clinic from the *Instituto Nacional de Infectologia Evandro Chagas (INI)*, *Fundação Oswaldo Cruz (Fiocruz)*, city of Rio de Janeiro, Brazil. The study was conducted before defining the etiological treatment, and the patients were classified as presenting Chagas cardiac or indeterminate clinical chronic forms at the beginning of the follow-up. Besides to investigate a possible association between parasite load, DTUs and severity of CCC, our results could reinforce the epidemiological knowledge of *T*. *cruzi* DTUs in Brazil.

## Methods

### Ethics, consent and permissions

This study was approved by the ethical committee of Fundação Oswaldo Cruz (CEP IPEC 007/2007), following the principles expressed in the Declaration of Helsinki. Chagas disease patients and healthy individuals participated as volunteers and agreed, giving a written consent, to the Free and Clarified Consent Terms.

### Study design

This work correspond to an observational, cross-sectional study, where the patients were recruited between 2011 to 2014 at the Chagas disease ambulatory from the *Instituto Nacional de Infectologia Evandro Chagas (INI—Fundação Oswaldo Cruz*, Rio de Janeiro, Brazil). As they were collected by convenience, there was no sample size calculation. To the individuals with epidemiological history of Chagas disease attended at INI, blood samples were collected and processed to perform the serological and molecular diagnosis tests. To the later, collected samples were splited in two, codified, and blinded delivered to the *Laboratório de Biologia Molecular e Doenças Endêmicas*, at the Oswaldo Cruz Institute/ Fiocruz. The inclusion criteria were: Two positive serological tests for Chagas disease (indirect immunofluorescence and ELISA), both genders, adult individuals (higher than 18 years old). The exclusion criteria were: patients with a previous treatment with benznidazole, co-infections, autoimmune disease, chronic diseases in which treatment requires the permanent use of inti-inflammatories or immunesupressors, patients with cancer and pregnants.

After the parasite load estimation by quantitative PCR, DNA samples were used to determine the *T*. *cruzi* genotyping. Results were pooled and analyzed according the characteristics of the patients.

### Patient samples

Sixty-five Chagas disease chronic patients, aged from 26 to 76 with a median of 54 years old (45–63), being 34 females and 31 males, were recruited. Following the Brazilian Consensus on Chagas Disease recommendations [32], patients were evaluated by electrocardiogram and classified accordingly to their clinical manifestations as chronic cardiac or indeterminate form of Chagas disease (Table 1). All patients presenting the chronic cardiac form have typical electrocadiographic alterations and were classified at stages A, B1, B2, C or D of the cardiomyopathy based on presence of heart failure and echocardiographic pattern. Each stage of A-D classification is associated with different prognosis, with a progressive increase in mortality. In this way, D patients with severe heart failure and systolic dysfunction have a 5-year mortality of 98%; in the stage C, patients with compensable heart failure have 91% of 5-year mortality; patients with no heart failure but systolic dysfunction on echocardiogram (B) have 5-year mortality of 45%; and finally, patients with no heart failure or systolic dysfunction, but with typical electrocardiographic alterations (A) have only 13% of 5-year mortality [33].

**Table 1:** General characteristics of patients included in the study.

General Characteristics		Clinical manifestation	
		Cardiac, n (%)	Indeterminate, n (%)
**Patients number**	65		
**Age, median [Q1 –Q3]**	54 [45–63]		
**Sex, n (%)**			
** Female**	34 (52.3)	16 (47.1)	18 (52.9)
** Male**	31 (47.7)	13 (41.9)	18 (58.1)
** Total**	65 (100)	29 (44.6)	36 (55.4)
**Region of birth, n (%)**			
** Northeast**	48 (73.8)	28 (58.3)	20 (41.7)
** Central West**	1 (1.6)	0 (0)	1 (100)
** Southeast**	14 (21.5)	4 (28.6)	10 (71.4)
** South**	2 (3.1)	0 (0)	2 (100)
** North**	0	0 (0)	0 (0)

Venous blood samples (10 mL) were collected from each patient, before etiological treatment, in EDTA-K2 vacutainer tubes (purple cap). Samples were immediately mixed with an equal volume of 6M Guanidine-HCl/0.2M EDTA solution (GEB) and then splitted in two identical aliquots. GEB samples were coded and blinded delivered to perform the molecular tests. At the laboratory, GEB samples were boiled for 15 minutes and stored at 4°C until use. Additionally, blood of individuals without *T*. *cruzi* infection (negative serology) from non-endemic Chagas disease areas were included as controls for DNA extraction and molecular assays. One negative control was used per DNA extraction of 11 GEB samples, and tested in parallel with the DNA samples in all qPCR assays.

### *T*. *cruzi* reference samples

Epimastigote forms of *T*. *cruzi* strains Dm28c (TcI), Y (TcII), INPA 3663 (TcIII), INPA 4167 (TcIV), and CL (TcVI) were obtained from the *Coleção de Protozoários da Fundação Oswaldo Cruz* (Colprot) and the clone Bug 2149 (TcV) was generously donated by Dr. Bianca Zingales (Instituto de Química, Universidade de São Paulo). Parasites were cultivated in brain-heart infusion (BHI) medium, supplemented with 16 mg/mL haemin and 10% (v/v) heat-inactivated fetal bovine serum, at 28°C. At the logarithmic phase of growth, cells were harvest by centrifugation and washed three times in phosphate-buffer saline (PBS) pH 7.4 before use.

### DNA extraction

DNA purification was performed using QIAamp DNA Mini Kit (Qiagen, Valencia, CA). This procedure was carried out as described by manufacturer’s protocol, with minor modifications, as follows: DNA was extracted from 300 μL Guanidine-EDTA blood samples (GEB) and, at the elution step, 100 μL of AE buffer were incubated with the silica-membrane column at room temperature for 10 min before DNA recovery, as described by Moreira et al. [27]. To the *T*. *cruzi* reference samples, GEB were spiked with the *T*. *cruzi* strains, boiled as mentioned, and DNA was extracted from 300 μL, as described.

### Molecular typing of *T*. *cruzi*

The genotyping of *T*. *cruzi* into 6 DTUs was performed following a combination of methodologies described by Ramírez et al. [16] and Burgos et al. [17], based on multilocus conventional PCR (Table 2). The PCRs targeted the intergenic region of Spliced Leader (SL-IR) [TCC, TC1 and TC2 primers, to distinguish between TcI (350 bp), TcII, TcV and TcVI (300 bp) and TcIII and IV (not amplified)], the D7 domain of the 24Sα ribosomal RNA gene [Heminested PCR: D75 and D76 (first round) and D76 and D71 (second round), to distinguish between TcII and TcVI (140 bp), TcIII (125 bp), TcIV (140/145 bp) and TcV (125 or 125+140 bp)] and the A10 nuclear fragment [Heminested PCR: Pr1 and P6 (first round) and Pr1 and Pr3 (second round), to differentiate TcII (690/580 bp) from TcVI (630/525 bp)]. The amplification reactions were performed in a Veriti Thermal Cycler (Applied Biosystems), as follows: 5 μL of extracted DNA were added to a 12,5 μL GoTaq Green Master Mix 2X (Promega, Madison, USA) containing GoTaq DNA polymerase, buffer (pH 8.5), 400 μM of each dNTP and 3 mM MgCl_2_, 1.25 μL of each primer (stock solutions: 25 μM for the SL-IR target, 10 μM for the 24Sα and A10 targets), and 5 μL of ultrapure water. PCR products (25 μL) were separated by agarose gel electrophoresis (2.5% w/v, 80V), stained with Nancy-520 (Sigma-Aldrich, St. Louis, USA) and visualized at UV light.

**Table 2:** Primers and conditions for *Trypanosoma cruzi* molecular typing.

Target	Primers	Sequence	Concentration	Termocycling			
				D	A	E	C
**SL-IR I and II**	TCC TC2 TC1	CCCCCCTCCCAGGCCACACTG CCTGCAGGCACACGTGTGTG TCCGCCACCTCCTTCGGGCC	2 µM 2 µM 2 µM	94°C/1’ 94°C/30’’ 94°C/1’ 94°C/1’	67°C/1’ 65°C/1’ 63°C/1’ 61°C/1’	72°C/1’ 72°C/1’ 72°C/1’ 72°C/1’	5 5 5 30
**24Sa-rDNA** **First round** **Second round**	D76 D75 D76 D71	GGTTCTCTGTTGCCCCTTTT GCAGATCTTGGTTGGCGTAG GGTTCTCTGTTGCCCCTTTT AAGGTGCGTCGACAGTGTGG	0,5 µM 0,5 µM 0,5 µM 0,5 µM	94°C/1’ 94°C/1’ 94°C/1’ 94°C/1’	60°C/1’ 57°C/1’ 55°C/1’ 58°C/1’	72°C/1’ 72°C/1’ 72°C/1’ 72°C/1’	5 5 5 35
**A10** **First round** **Second round**	Pr1 P6 Pr1 Pr3	CCGCTAAGCAGTTCTGTCCATA GTGATCGCAGGAAACGTGA CCGCTAAGCAGTTCTGTCCATA CGTGGCATGGGGTAATAAAGCA	0,5 µM 0,5 µM 0,5 µM 0,5 µM	94°C/1’ 94°C/1’ 94°C/1’ 94°C/1’ 94°C/1’	60°C/1’ 57°C/1’ 55°C/1’ 58°C/1’ 65°C/1’	72°C/1’ 72°C/1’ 72°C/1’ 72°C/1’ 72°C/1’	5 5 5 35 35

**D:** denaturation, **A:** annealing, **E:** extension, **C:** cycles.

DNA extraction was performed from two aliquots of the same GEB sample. The genotyping was performed in parallel, and results were pooled, as showed in Table 3.

**Table 3:** Molecular typing of *T*. *cruzi* from patient blood samples. Samples A and B correspond to the DNA duplicates from the same patient, used for the genotyping assays. According to the genotyping flowchart (Fig 3), X represents the *T*. *cruzi* genotype in each sample.

Patient	Samples	DTU						*T*. *cruzi* genotype
		II	III	V	VI	II/VI	II/V/VI	
1	A	X						II
	B	X						
2	A	X						II
	B	X						
3	A	X						II
	B	X						
4	A					X		II
	B	X						
5	A	X						II
	B							
6	A							II
	B	X						
7	A							II/V/VI
	B						X	
8	A						X	II/V/VI
	B						X	
9	A						X	II/V/VI
	B						X	
10	A					X		II/VI
	B							
11	A							II/VI
	B					X		
12	A							II/VI
	B					X		
13	A							II/VI
	B					X		
14	A	X			X			II+VI
	B					X		
15	A				X			II+VI
	B	X			X			
16	A	X						II+VI
	B	X			X			
17	A				X			II+VI
	B	X			X			
18	A							II+VI
	B	X			X			
19	A				X			III+VI
	B		X					
20	A							V
	B			X				
21	A			X				V
	B							
22	A						X	VI
	B				X			
23	A				X			VI
	B					X		
24	A					X		VI
	B				X			
25	A				X			VI
	B				X			
26	A							VI
	B				X			
27	A					X		VI
	B				X			
28	A							VI
	B				X			
29	A				X			VI
	B							
30	A				X			VI
	B					X		
31	A				X			VI
	B							
32	A				X			VI
	B							
33	A				X			VI
	B							
34	A				X			VI
	B							
35	A				X			VI
	B							

### Parasite load quantification

Multiplex qPCRs were carried out as described by Pirón et al. [25], using TaqMan probes targeting *T*. *cruzi* nuclear satellite DNA (FAM/NFQ-MGB) and human RNAse P gene (VIC/ TAMRA), with the following modifications: 5 μL DNA were mixed with 10 μL Fast Start Universal Probe Master Mix (Rox) (2X) (Roche, Basel, Switzerland), 300nM Cruzi1 (5’- AST CGG CTG ATC GTT TTC GA- 3´) and Cruzi2 (5´- AAT TCC TCC AAG CAG CGG ATA- 3´) primers and 100nM Cruzi3 probe (5’-FAM CAC ACA CTG GAC ACC AA- NFQ-MGB-3’), targeting the satellite region of the nuclear DNA of *T*. *cruzi*; with the addition of 0,5X TaqMan Human RNase P detection reagent Kit (VIC/TAMRA—Applied Biosystems), in a final volume of 20 μL. Cycling conditions were a first step at 95°C for 5 min, followed by 40 cycles at 94°C for 15 sec and at 58°C for 1 min. The amplifications were carried out in an ABI Prism 7500 Fast device (Applied Biosystems, USA). The threshold was set at 0.02 for both targets. For the absolute quantification by real time qPCR, standard curves were constructed by serial dilution of DNA extracted from GEB sample spiked with *T*. *cruzi* epimastigotes (CL Brener), ranging from 10^4^ to 0.5 parasite equivalents per milliliter of blood (par. Eq./mL), as described [23]. The parasite load was quantified in all GEB samples and expressed as the average between the duplicates for each patient. The DNA quality and absence of PCR inhibition was monitored by the Ct value for the RNAse P target, expected in a range between 24–25.

### Statistical analysis

All experiments were performed at least in two technical replicates. Categorical data were expressed as percentages. Continuous data were expressed as means ± standard deviation or median and interquartile range, according the normality of distribution. Student’s t-test or Mann–Whitney Rank Sum test was used to analyze the statistical significance of the observed differences. A p-value of less than 0.05 was considered statistically significant. Analyses were performed with SigmaPlot for Windows version 12.0 (Systat Software, Inc).

## Results

### Characteristics of the patients included in this study

In this single-blind study, sixty-five chronic patients assisted between 2011 to 2014 at the Chagas disease ambulatory from the *Instituto Nacional de Infectologia Evandro Chagas (INI—Fundação Oswaldo Cruz*, Rio de Janeiro, Brazil) were included, as described in Table 1. All patients presented positive serology for *T*. *cruzi* and were migrants from different geographical regions from Brazil. Due to the birthplace in endemic areas and to have lived in houses with wattle and daub walls or to have previous contact with the triatomine vector, the most probable cause of CD was through vectorial transmission.

From the 65 patients, 36 presented the chronic indeterminate form and 29 presented the chronic Chagas heart disease, and were previously classified at the stage A (15 patients), B1 (7 patients) and C (7 patients). The median of age was 54 [45–63] years. A total of 34 females and 31 males presented the cardiac form of the disease (47.1% and 41.9%, respectively). Most of patients were migrants from the northeast region of Brazil (48), followed by the southeast region (14). In this study, no patient from the North region of the country was recruited.

### Parasite load

**Fig 1:**
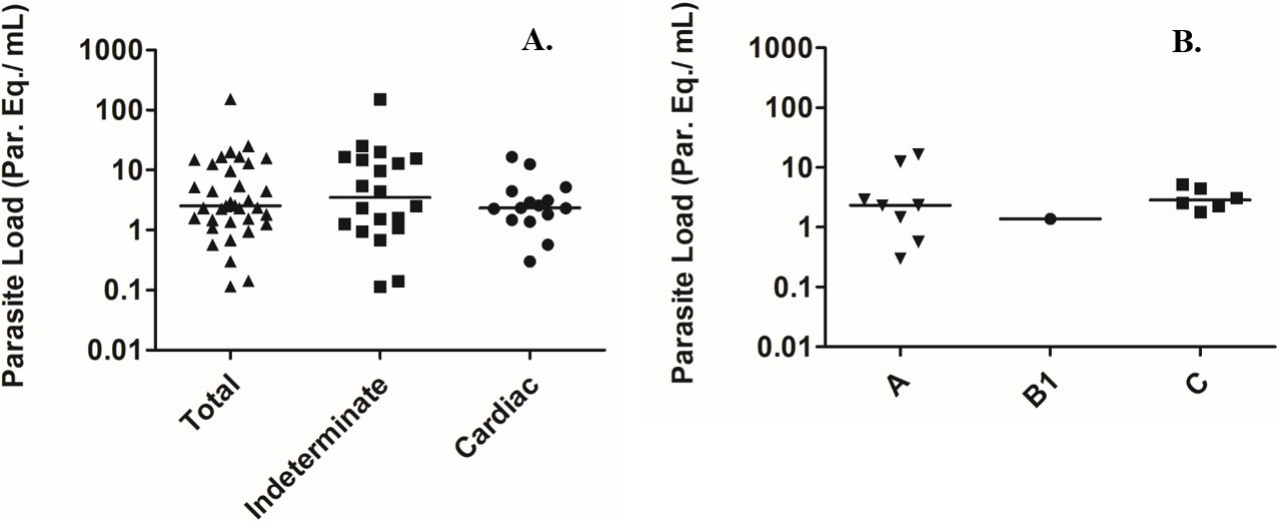
Parasite load in chronic patients with different clinical manifestations of Chagas disease. In A, total parasite load (▲) and according the type of clinical manifestation: indeterminate (■) or chronic Chagas heart disease (●). In B, parasite load according the severity of the cardiomyopathy: A (▼), B1 (●) and C (■). The horizontal lines represent the median values of the parasite load.

Of the 65 patients, 35 (53.8%) presented positive amplification by real time PCR targeting the *T*. *cruzi* nuclear satellite DNA, being 20 (30.7%) with the indeterminate form and 15 (23.1%) with the chronic Chagas heart disease. The parasite load median from all positive patients was 2.54 [1.43–11.14] par. Eq./mL, with the load ranging from 0.12 to 153.66 par. Eq./mL. To the patients with the indeterminate form, a median of 3.49 [1.22–15.10] par. Eq./mL was observed and to the patients presenting the chronic Chagas heart disease, a median of 2.34 [1.65–3.78] par. Eq./mL was observed (Fig 1A). When the parasite load was compared among patients with distinct levels of cardiomyopathy severity (Fig 1B), classified according to the score established by the Brazilian Consensus for Chagas disease, the median values were 2.33 [1.25–5.32] and 2.85 [2.35–4.11] par. Eq./mL for the levels A and C, respectively. Only one patient with cardiomyopathy was classified in the B1 level, presenting 1.39±0.96 par. Eq./mL. It was also observed no significant statistical difference in the parasite load between the levels of cardiomyopathy A or C. The statistical analysis was not performed to the B1 group because of the sample size. Even observing differences in the parasite load between those two groups, no statistically significant difference was observed.

**Fig 2:**
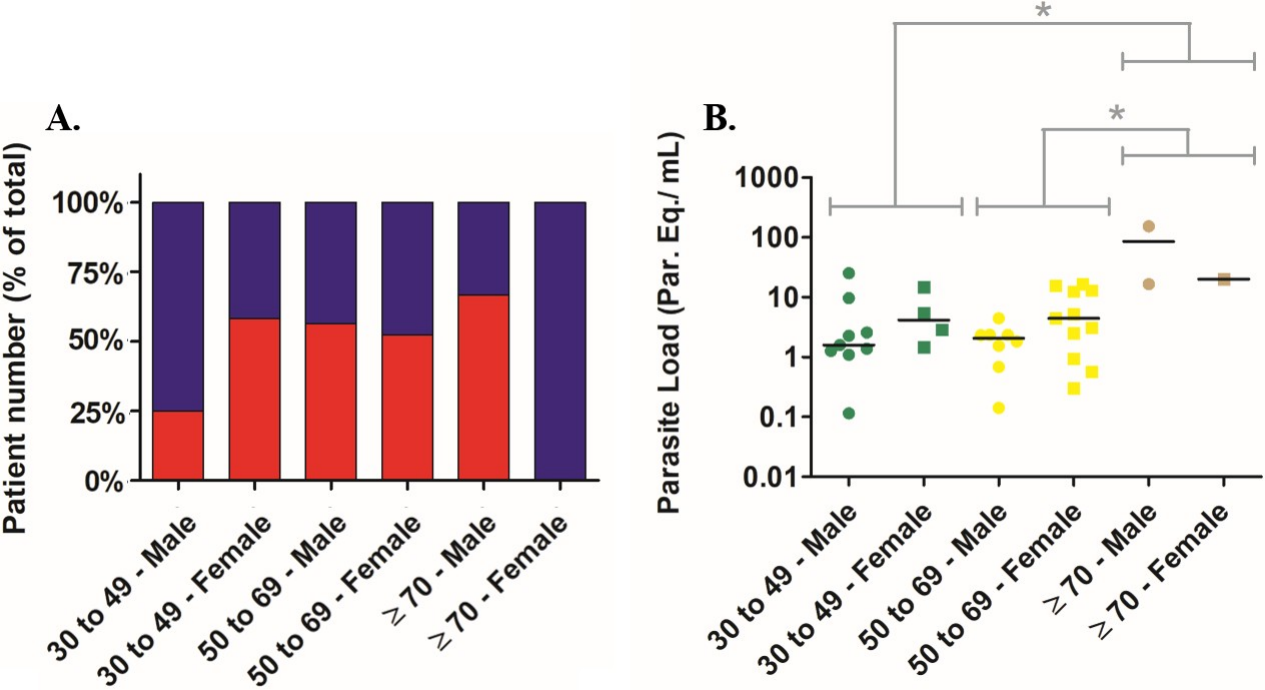
Clinical manifestations and parasite load in chronic patients grouped by age and gender. In A, clinical manifestations grouped by patients age and gender. Red bars: patients with cardiac form (CCC). Blue bars: patients with indeterminate form. In B, parasite load in blood of patients grouped by age and gender: Green symbols: from 30 to 49 years old; Yellow symbols: from 50 to 69 years; Brown symbols: over 70 years old. (●) Male patients, (■) Female patients. The horizontal lines represent the parasite load median values. *P<0.05 (Mann-Whitney Rank Sum test).

To analyze the patients according to the gender and age simultaneously, they were grouped from 30 to 49 years, 50 to 69 years and ≥ 70 years (Fig 2A). In the 30 to 49 years old group (24 patients: 12 males and 12 females), 3 (25%) and 9 (75%) male patients presented the cardiac (CCC) and indeterminate form of CD, respectively. In the same group, 7 (58.3%) and 5 (41.7%) female patients presented the cardiac (CCC) and indeterminate form of CD, respectively. On the other hand, in the 50 to 69 years old group (37 patients: 16 males and 21 females), 9 (56.3%) and 7 (43.7%) male patients presented the cardiac (CCC) and indeterminate form of CD, respectively. In the same group, 11 (52.4%) and 10 (47.6%) female patients presented the cardiac (CCC) and indeterminate form of CD, respectively. At last, to the ≥ 70 years old group (4 patients: 3 males and 1 female), 2 (66.7%) and 1 (33.3%) male patients presented the cardiac (CCC) and indeterminate form of CD, respectively. In the same group, none and 1 female patients presented the cardiac (CCC) and indeterminate form of CD, respectively.

The parasite load could be estimated to 19 males and 16 females (Fig 2B). In the 30 to 49 years old group, the parasite load medians from male and female patients were 1.59 [1.18–6.14] and 4.17 [1.83–12.5] par. Eq./mL, respectively. To the 50 to 69 years old group, the parasite load medians from male and female patients were 2.07 [0.90–2.36] and 4.45 [0.95–13.07] par. Eq./mL, respectively. To the ≥ 70 years old group, the parasite load median from male patient was 85.09 [16.52–153.70] par. Eq./mL. On the other hand, there was only one female patient over 70 years old, with a parasite load of 20.05 ± 2.75 par. Eq./mL. Although no statistical difference could be observed between males and females, higher parasite load medians were observed to the females from the 30 to 49 and 50 to 69 years old groups. Remarkably, regardless the gender, the comparison between the age groups showed that the parasite load was significantly higher in patients over 70 years old (median 20.05 [18.29–86.86] par. Eq./mL) in comparison to patients between 30 and 49 (median 2.26 [1.39–5.44] par. Eq./mL) or 50 to 69 years old (median 2.37 [1.25–4.81] par. Eq./mL).

### Molecular typing of *T*. *cruzi*

#### Standardization of T. cruzi genotyping and establishment of a reference strains panel

**Fig 3:**
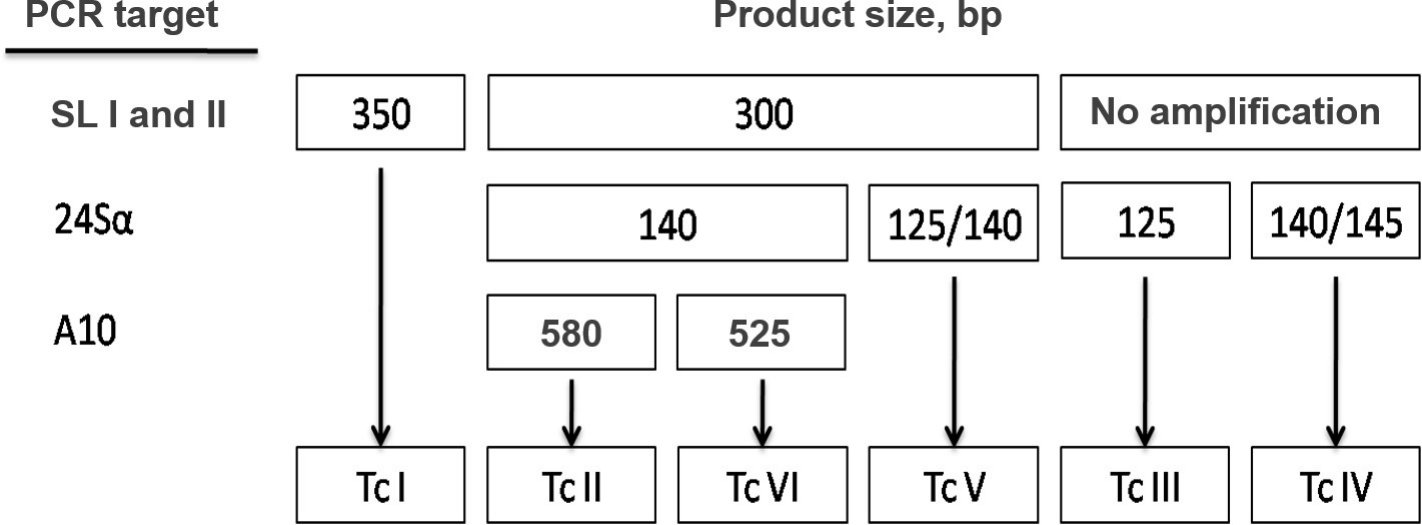
Multilocus conventional PCR flowchart for *T*. *cruzi* genotyping directly from human blood samples, based on three molecular markers. The flowchart indicates PCR product size in bp. SL-IR I and II: spliced-leader intergenic region I and II. 24Sα: heminested amplification of the D7 domain of 24Sα ribosomal RNA genes. A-10: heminested reaction for the A-10 fragment.

The molecular typing of *T*. *cruzi* was performed by multilocus conventional PCR targeting the intergenic region of Spliced Leader (SL-IR I and II), the D7 domain of the 24Sα ribosomal RNA gene and A10 nuclear fragment [16, 17], according to the flowchart described in Fig 3. In order to improve the sensitivity and specificity for human blood samples, the PCR conditions were slighty modified, as described in the Methodology section. To perform the experiments, reference strains/clones of *T*. *cruzi* obtained from axenic cultivation were used, as follows: Dm28c (TcI), Y (TcII), INPA 3663 (TcIII), INPA 4167 (TcIV), Bug2149 (TcV) e CL (TcVI). *T*. *cruzi* genotypes were distinguished by the sizes of PCR product visualized in stained agarose gels (Fig 3).

**Fig 4:**
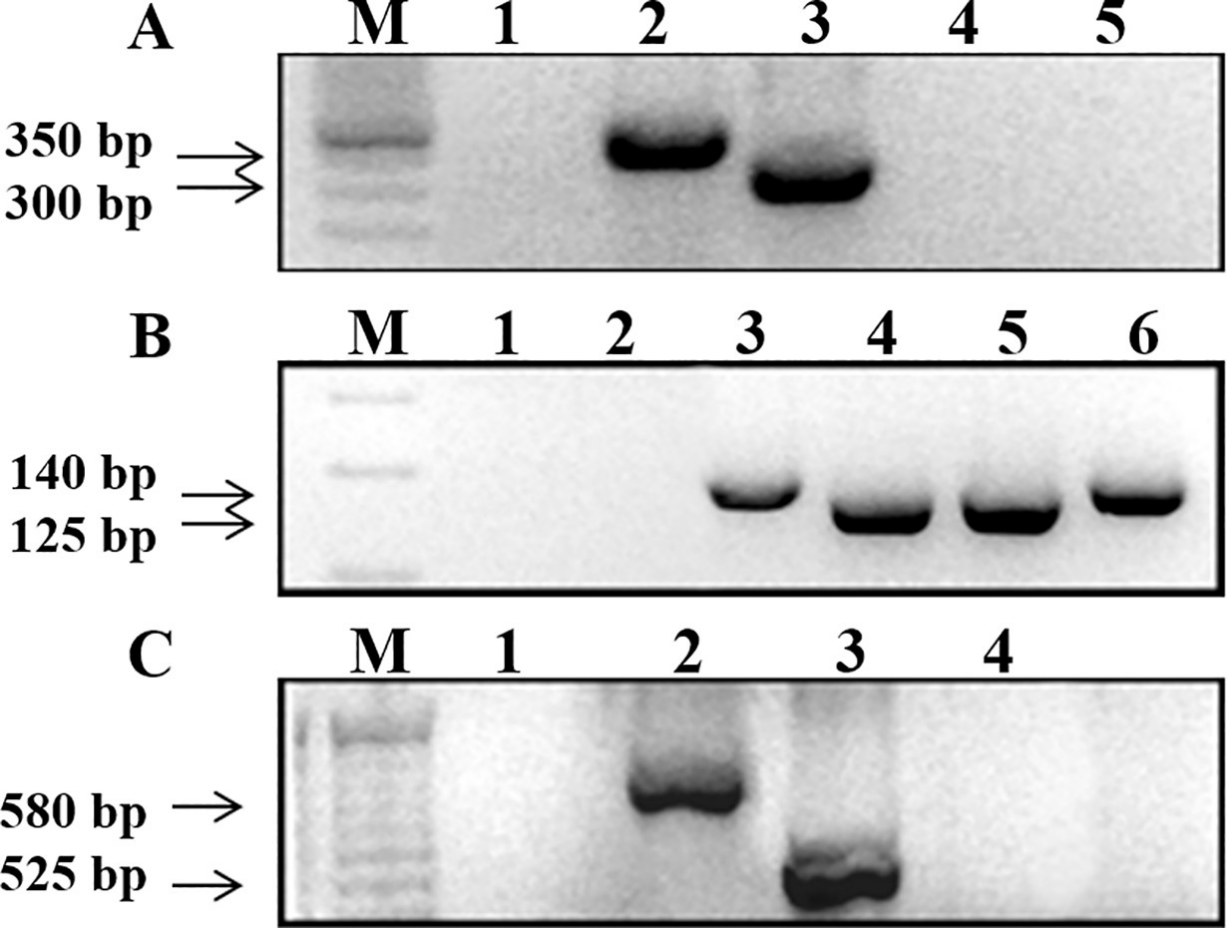
Multilocus conventional PCR for *T*. *cruzi* genotyping with the reference strains/clones. PCR product sizes are indicated on the left by arrows. In A, SL-IR I and II target: Lanes—1: negative control; 2: Dm28c clone (TcI); 3: Y strain (TcII); 4: INPA 4167 strain (TcIV); 5: no template control (NTC). In B, 24Sα (second round) target: Lanes—1: NTC; 2: negative control; 3: Y strain; 4: INPA 3663 strain (TcIII); 5: Bug 2149 clone (TcV); 6: CL strain (TcVI). In C, A-10 target: 1: NTC, 2: Y strain (TcII), 3: CL strain (TcVI), 4: negative control. M- DNA ladder (100 or 50 bp).

For the intergenic region of spliced leader gene (SL-IR I and II), it was possible to distinguish between TcI with 350 bp PCR product size from TcII, TcV and TcVI with 300 bp, while TcIII and TcIV did not show amplification. Using a hemi-nested PCR targeting 24Sα ribosomal subunit, it was possible to differentiate TcV with a fragment between 125/140 bp from TcII and TcVI both with 140 bp. Moreover, TcIII showed a 125 bp fragment and for TcIV the PCR product size was between 140/145 bp. Despite the similarity of amplicon sizes, it was possible to differentiate from the other DTUs when analyzed in conjunction with the first marker. To discriminate TcII from TcVI (both generated fragments with 140 bp in the 24Sα ribosomal PCR), a hemi-nested conventional PCR for the A10 nuclear fragment was used, and the product sizes were 580 bp (TcII) and 525 bp (TcVI). Fig 4 shows representative images of 1% (w/v) agarose gels containing PCR products from *T*. *cruzi* reference strains/clones, indicating the expected fragment sizes. In all PCRs, no template controls (NTCs) and negative controls (human blood DNA from patients with negative serology for Chagas disease) were used, and it was not observed any amplified fragment with molecular weight similar to the *T*. *cruzi* expected ones.

In order to confirm the PCR specificity, DNA sequences were obtained and compared to sequences from GeneBank. The 300 bp and 350 bp fragments (SL IR I and II), 125 bp and 140 bp (24Sα) and 525 bp and 580 bp (A10) were excised from the gels, purified and submitted to DNA sequencing. All sequences were submitted to BLASTn alignment and confirmed with a query cover higher than 97% and identity higher than 88% (S1 Table).

**Fig 5:**
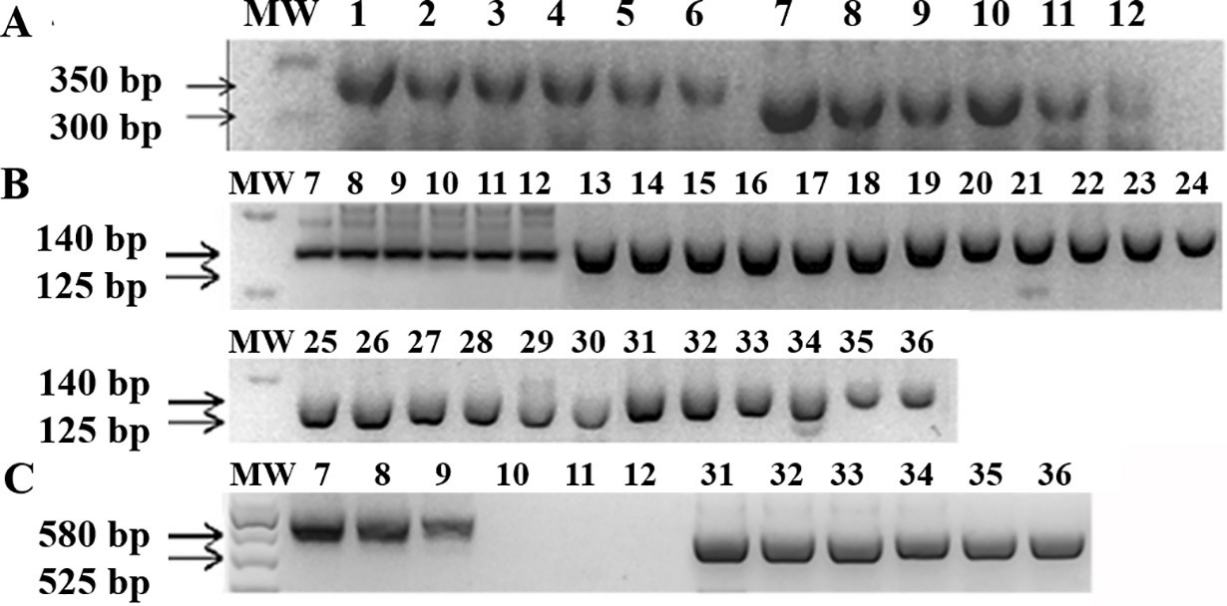
Analytical sensitivity of multilocus conventional PCR for *T*. *cruzi* genotyping from spiked blood samples. The PCR product sizes are indicated on the left by arrows. In A, SL-IR I and II target. In B, 24Sα (second round) target. In C, A-10 target. MW: DNA ladder (100 or 50 bp). Lanes 1–6: Dm28c clone (TcI); 7–12: Y strain (TcII); 13–18: INPA 3663 strain (TcIII); 19–24: INPA 4167 strain (TcIV); 25–30: Bug 2149 clone (TcV); 31–36: CL strain (TcVI). For all samples, Guanidine-EDTA blood was spiked with *T*. *cruzi* ranging from 10^4^ to 0.5 par. Eq./mL (10^4^, 10^3^, 10^2^, 10, 1 and 0.5 par. Eq./mL) before DNA purification.

To evaluate the analytical sensitivity for *T*. *cruzi* genotyping, guanidine-EDTA blood samples were spiked with parasites from the reference strains/clones, ranging from 10^4^ to 0.5 par. Eq./mL prior to DNA purification. PCRs were performed as described, resulting in PCR products with the expected sizes for each target (Fig 5). Throughout the analysis of the obtained PCR products, it was possible to differentiate DTUs from blood samples with high sensitivity: for the SL IR I and II and 24Sα markers (Fig 5A and 5B), all samples were successfully amplified up to 0.5 par. Eq./mL. Nevertheless, for the A10 target (Fig 5C), blood sample spiked with Y strain (TcII) only amplified up to 10^2^ par. Eq./mL. On the other hand, for the same target, sample spiked with CL strain (TcVI) successfully amplified the 525 bp PCR product in all tested concentrations.

### *T*. *cruzi* genotyping from blood samples of chronic Chagas disease patients

**Fig 6:**
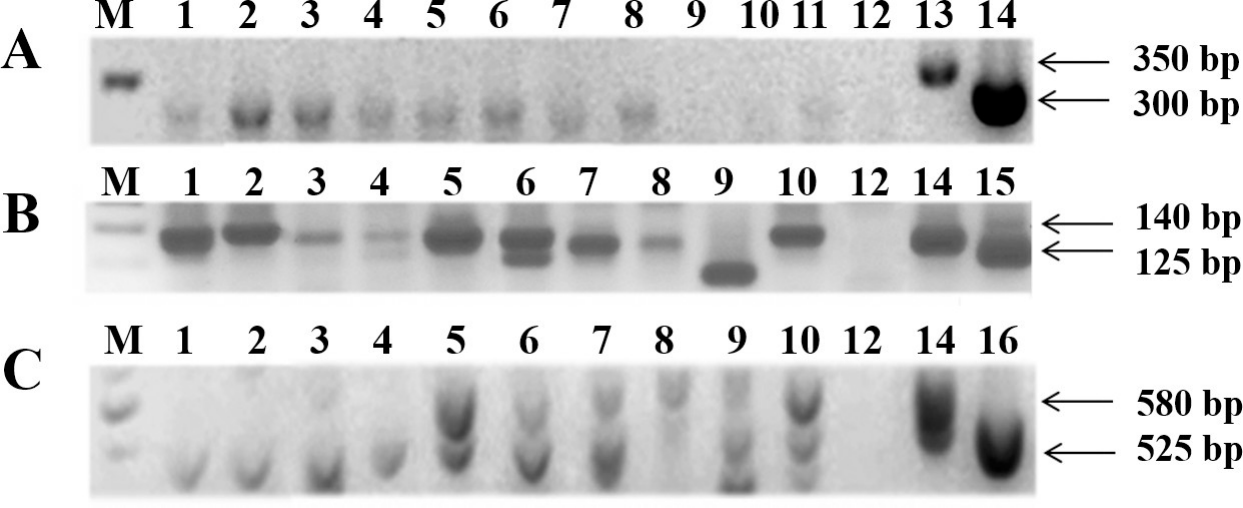
Multilocus conventional PCR for *T*. *cruzi* genotyping from patient blood samples. PCR product sizes (bp) are indicated on the right by arrows. In A, SL-IR I and II target. In B, 24Sα (second round) target. In C, A-10 target. Lanes 1–11: patient samples. Lane 12: no template control (NTC), Lane 13: Dm28c clone (TcI), Lane 14: Y strain (TcII), Lane 15: INPA 3663 strain (TcIII), Lane 16: CL strain (TcVI). M: 50 or 100 bp DNA ladder.

The molecular typing of *T*. *cruzi* was performed in samples from the 35 patients with positive results for the real time PCR assays targeting nuclear satellite DNA. In order to increase the sensitivity, genotyping was performed in duplicates (samples A and B in Table 3), using the same DNA samples for the qPCR assays, and the results were analyzed in conjunction. This approach allowed us to determine the *T*. *cruzi* genotypes of all patients, and pointed out the mixed infections precisely. Furthermore, just one patient presented a discrepant result between the duplicates, indicating the good reproducibility and efficacy of this methodology. Fig 6 shows representative images of agarose gels with positive amplifications for SL-IR I and II, 24Sα rDNA and A10 targets.

As shown in Table 3, the most abundant DTU found was TcVI (14), followed by TcII (6) and TcV. (2). Interestingly, we found mixed infections in five patients with TcII+TcVI and one case with TcIII+TcVI. However, three patients yielded positive amplification with 300 bp for SL-IR I and II but negative for 24Sα rDNA, being reported as TcII/V/VI. Additionally, four patients positively amplified with 300 bp for SL-IR I and II and with 140 bp for 24Sα rDNA, but presented negative results for A10 target, in only one replicate sample. These patients were reported as infected by TcII/VI.

### Geographic distribution of the *T*. *cruzi* DTUs

This study was composed by individuals from all regions of Brazil, except the North region, which comprises the Amazon forest. Patients were born in rural zones or very small cities, in endemic areas. At least during the childhood, they declared to have lived in houses with wattle and daub walls or to have contact with the triatomine vector. When adults, most of these individuals migrated to the Rio de Janeiro city, which is not endemic to Chagas disease. So, it is very probable that they were infected by the triatomine vector, during the childhood.

Of all positive samples, TcII and TcVI were the most prevalent detected DTUs. They were also the most geographically disperse DTUs: in single or mixed infections, TcII was present in six States of Brazil (Ceará, Paraíba, Pernambuco, Alagoas, Minas Gerais and Rio de Janeiro) while TcVI was present in eight States (Ceará, Paraíba, Pernambuco, Alagoas, Bahia, Goiás, Minas Gerais and Rio de Janeiro), respectively (Table 4). Interestingly, two cases of infection by TcV and one case of mixed infection by TcIII+TcVI (with the cardiac form of the disease) were identified in the Bahia State, at the Northeast region of Brazil. The Ceará State presented the highest prevalence of the cardiac form of the disease (5/7 patients– 71.4%), associated to TcII and TcVI. At the Sergipe State, the only patient with positive detection and genotyping of *T*. *cruzi* (TcVI) presented the cardiac form of the disease. No TcI genotype was identified in this study. These results highlight the current dispersion and variability of *T*. *cruzi* DTUs in Brazil.

**Table 4:** Geographic distribution of *T*. *cruzi* DTUs in the patient samples analyzed.

Place of birth (Region and state)	Number of patients	*T*. *cruzi* genotype	Clinical Manifestation
**Northeast**			
** Ceará**	2 1 1 1 2	TcII TcVI TcII/VI TcII/V/VI TcII+TcVI	cardiac, indeterminate cardiac cardiac Indeterminate cardiac, cardiac
** Paraíba**	3 1	TcVI TcII+TcVI	indeterminate, cardiac, indeterminate cardiac
** Pernambuco**	1 1 1	TcII TcVI TcII/VI	Indeterminate indeterminate cardiac
** Alagoas**	1 1 1 1	TcVI TcII/VI TcII/V/VI TcII+TcVI	Cardiac indeterminate indeterminate indeterminate
** Sergipe**	1	TcVI	Cardiac
** Bahia**	2 4 1	TcV TcVI TcIII+TcVI	cardiac, indeterminate indeterminate, cardiac, indeterminate, cardiac cardiac
**Central West**			
** Goiás**	1	TcVI	Indeterminate
**Southeast**			
** Minas Gerais**	2 2 1	TcII TcVI TcII/VI	indeterminate, indeterminate indeterminate, cardiac indeterminate
** Rio de Janeiro**	1 1	TcII/V/VI TcII+TcVI	indeterminate indeterminate
**South**			
** Rio Grande do Sul**	1	TcII	Indeterminate

### Association between clinic manifestation, DTUs and parasite load

**Fig 7:**
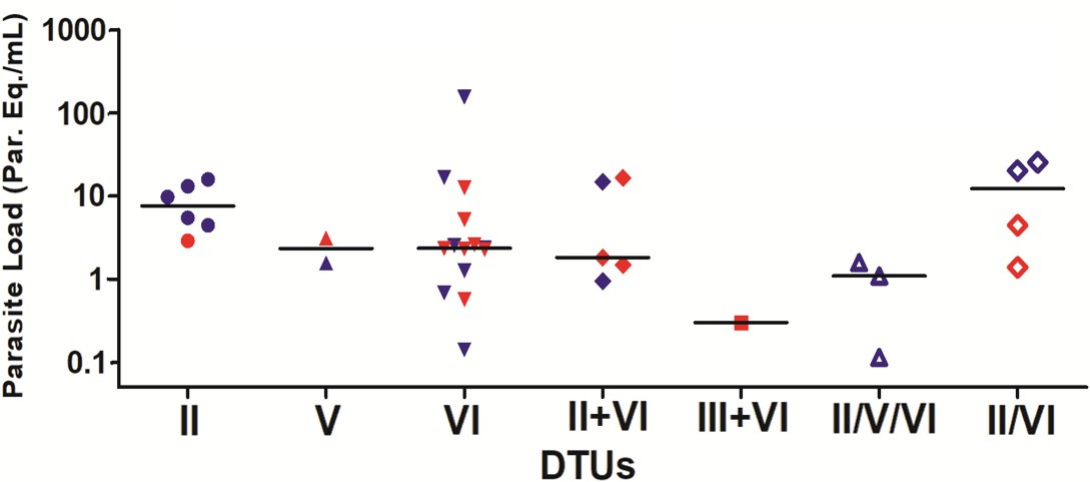
Comparison between *T*. *cruzi* genotype, clinical form and parasite load. The symbols represent the parasite load for each patient, distributed by *T*. *cruzi* DTUs. The horizontal lines correspond to the median values of parasite load in each DTU. Red symbols indicate patients with CCC and blue symbols indicate patients with the indeterminate form.

*T*. *cruzi* genotypes circulating in the peripheral blood were also compared between patients with or without chronic Chagas disease cardiomyopathy (CCC), as showed in Fig 7. The frequency of chronic Chagas heart disease was the same of the chronic indeterminate form in patients infected by TcVI (seven patients with and without CCC). Although there was only two patients infected with TcV, no difference was also observed in their clinical forms (one patient with CCC and one with indeterminate form). However, between the six patients infected by TcII, five (83.3%) presented the indeterminate form and only one (16.7%) presented CCC. Nevertheless, when TcII occurred together TcVI (five patients coinfected) the frequency of CCC increased to 60% (3 patients). In the only patient coinfected with TcIII and TcVI, the clinical form described was CCC. For three patients, all with the indeterminate form, it was not possible to distinguish between TcII, V and VI. In addition, relative to four patients where it was not possible to distinguish between TcII or TcVI, there were two cases of CCC (50%).

*T*. *cruzi* genotypes were also compared versus the parasite load for each patient (Fig 7 and S2 Table), both in chronic indeterminate form and CCC. In general, a higher parasite load was observed in patients infected by TcII, with a median of 7.56 par. Eq./mL. Patients infected by TcVI showed a broader parasite load range, from 0.14 to 153.66, with median of 2.35 par. Eq./mL. Patients with co-infection by TcII and TcVI present a similar parasite load median to the group TcVI (1.82 par. Eq./mL). For the group of patients infected by the other DTUs, in single or co-infections, the number of specimens was too small to observe some parasite load profile, although higher parasite load was observed in the group where it was not possible to differentiate between TcII and TcVI (TcII/TcVI: median parasite load = 12.24 par. Eq./mL).

When the parasite load was compared between patients infected by TcVI with or without CCC, the observed parasite load medians were, respectively, 2.34 and 1.27 par.Eq./mL. Although the TcVI-patients with Chagas cardiomyopathy have presented higher parasite load, no statistically significant difference was observed.

## Discussion

The relevance of parasite load and *T*. *cruzi* genotype as biomarkers of CD prognosis still represents a big challenge. The genetic diversity of the parasite should interfere in the pathogenesis of the disease, in conjunction with the host immune response profile and intensity. However, at the present time, few studies were able to support this premise for the chronic patients. Herein, an intraspecific multilocus *T*. *cruzi* genotyping approach and a pre-established and validated qPCR protocol were used, as recommended by the international consensus of specialists in this field [15, 17, 25, 27, 28], enabling the molecular characterization and parasite load quantification directly from blood samples of chronic patients, with a high sensitivity. Those parameters could provide information about the correlation between the main subgroups of *T*. *cruzi* and clinical aspects of the infection. Accordingly, from an urban cohort of patients born in different regions of Brazil, we afforded new information of the complexity and dynamics of parasite diversity in the natural history of human CD.

This study comprised 65 patients with chronic CD. The parasite load was compared between individuals with or without CCC, presenting different levels of cardiomyopathy progression, gender and age. As expected for chronic patients from Brazil, the parasite load was low [27, 28, 29], and some patients presented parasitemia below the limit of quantification previously published [34]. Regarding patient gender and age, no statistical difference was observed in parasite load between groups, although female patients in the 30 to 49 years old group presented a tendency to higher parasite load, and higher heart disease onset frequency. In the 50 to 69 years old group, even with the same tendency to higher parasite load attributed to the females, no difference was observed in the frequency of CCC between males and females. On the other hand, regardless the gender, the group of patients older than 70 years presented a remarkable higher parasitemia levels. The oldest patient with detectable parasite load in this study, with 74 years old, had a previous history of myocardial infarction and chronic renal failure, both associated to *diabetes mellitus*. He presented the highest parasite load: 153.66 ± 27.49 par. Eq./mL. This is a noteworthy difference. One of the hypotheses for this finding could be the presence of the comorbidities, or the advanced age itself, that could lead to an immunosuppression associated to CD reactivation. In these cases, the parasite load could be used as biomarker of reactivation.

Despite the discovery of several molecular markers over the years, there is still no solely one capable to genotype *T*. *cruzi* into the 6 DTUs or TcBat. Thus, the multilocus molecular typing by PCR, PCR-RFLP or DNA sequencing are the most reliable methods to identify the parasite genotype [5]. In the present study, *T*. *cruzi* genotyping was performed based on an algorithm of molecular markers developed from the combination of two previously published methodologies [16,17]. Moreover, the molecular characterization was performed from DNA extracted directly from blood samples, to avoid a possible selection of strains during parasite isolation, which could not detect mixed infections in the specimens. As shown by Moreira et al. [27], the parasite burden of patients with CCC in Brazil was lower when compared with patients from other endemic countries, suggesting the need of an optimized protocol for *T*. *cruzi* genotyping from blood samples. In fact, our genotyping results showed a high sensitivity: it was possible to visualize PCR products with the expected sizes for each target in agarose gels, using DNA extracted from GEB samples spiked with *T*. *cruzi* from 10^4^ up to 0.5 par. Eq./mL, which is consistent with the parasite load found in Brazilian patients in the chronic phase of the disease. Except for the A10 target, where the limit of detection for TcII (580-bp fragment) was 10^2^ par. Eq./mL. This is in agreement with the results previously reported [35], where the sensitivity for TcII b (current TcII) was 10 pg of *T*. *cruzi* DNA, 10 times lower than the one observed for TcII e (current TcVI)– 1 pg of *T*. *cruzi* DNA. Besides, the DNA sequences corresponding to each PCR product excised from the gels confirmed the identity of the expected products generated for the genomic *T*. *cruzi* targets used in this multilocus PCR approach (S1 Table), showing a high specificity of the methodology. In an attempt to genotype a high percentage of samples, we extracted DNA twice from each patient GEB sample. *T*. *cruzi* genotyping was performed in parallel (samples A and B, in Table 3) and the results were compared. Of the 35 patients with qPCR positive, it was possible to accurately identify the *T*. *cruzi* DTUs in 28 (80%). For the remaining patients (20%), at least a partial result was obtained. In conjunction, this methodology obtained a high success rate in genotyping *T*. *cruzi* from CD chronic patients.

The previous knowledge about DTUs in Chagas disease patients points out the prevalence of TcII and TcVI within the area of vectorial domestic transmission in Brazil [5, 19]. In a recent study with nine patients from Pernambuco (PE), Paraíba (PB), Bahia (BA), Minas Gerais (MG), and Rio Grande do Sul (RS) states [36], also assisted at the outpatient clinic from the *Instituto Nacional de Infectologia Evandro Chagas (INI)* at *Fundação Oswaldo Cruz (Fiocruz)*, *T*. *cruzi* was isolated from blood samples and the DTUs were identified. A high prevalence of TcII (66.7%) followed by TcVI (22.2%) was observed. In addition, one patient infected by TcI was identified (11.1%). Nevertheless, in 2014, a review was published based on data from 1980 to 2012 [37], reporting the genetic diversity of *T*. *cruzi* beyond DTUs TcII and TcVI. Part of the Brazilian states analyzed, such as CE, BA, PE, PA, MG and GO, corresponded to the same origin of some individuals in our study, and the DTUs reported here are in agreement with the observed prevalence. In addition, our data extend the genetic diversity of the parasite by presenting cases of *T*. *cruzi* infection in the states of AL and RJ. Despite controlling the transmission by the main vector species, a considerable prevalence of CD is still found in some Brazilian areas, with a decline in recent decades. Herein, a prevalence of TcVI, TcII and mixed infection by TcVI+TcII (occurring in 40%, 17.1% and 14.3% respectively–Table 3), in patients widely distributed across the geographic regions of the country, was observed (Table 4). This high prevalence of TcVI and TcII in Brazil is partially in agreement with the literature [5, 19], which suggests the wide distribution of TcII in the domestic cycle of CD, from Northeast to Southern Brazil, and TcVI throughout the Southeast and South, including the Midwest region. However, nine cases of infection by TcVI were observed at the Northeast region. This finding, together with the observed TcII+TcVI coinfections, represents the intersection of epidemiological areas for these two DTUs or an underestimation of this distribution. In addition, the identification of patients with coinfection by two different DTUs emphasizes the importance of molecular typing directly from the blood when compared with classical methods for the isolation of parasites [38,39].

Interestingly, cases of human infection by TcV in Brazil had not been identified to date. Nevertheless, in this study, two patients infected by TcV were identified in the Bahia state (Northeast region). This is in agreement with the recent identification of TcV in reservoirs in the Brazilian Amazon [40]. These results indicate that the transmission area of TcV may be broader than is known, highlighting the importance of the main DTU correlated with *T*. *cruzi* infection in the Argentinean Chaco and Bolivia [38]. Recently, infection by TcIII in three chonic CD patients was associated with the indeterminate form of the disease, in the semiarid region of Brazil [22]. In contrast, we found a patient from the state of Bahia presenting the stage A of cardiomyopathy, coinfected by TcIII plus VI, pointing out the possible association between TcIII genotype and CCC. Moreover, in this study, the parasite load was estimated from the same DNA sample used to determine the *T*. *cruzi* genotype. By analyzing those data in accordance with the presence or absence of heart disease onset, we could not observe any association, as we previous reported in another study [29], regardless the *T*. *cruzi* genotype identified in the blood samples (Fig 7). Noteworthy, even considering the small and different number of samples between the group of patients infected by TcII and TcVI, increased parasite loads were observed to patients with TcII in general, but with lower observance of CCC (occurring in only 16.7%). This observation raises some questions that should be further addressed in a new study, containing a high number of individuals and equal sampling size between DTU groups, in order to perform a more reliable comparison.

In conclusion, our experiments reinforce the epidemiological knowledge of *T*. *cruzi* DTUs in Brazil, in special for the prevalence of TcII and TcVI, but also indicating that TcIII and TcV transmission areas may be underestimated. Additionally, considering the number of patients enrolled in this study, the age of patient and infection by TcII seems to be important features that lead to the observation of higher parasitemia levels.

## Supporting information

S1 Table DNA sequences identification from PCR products for SL-IR I and II, 24Sα and A10 targets. (DOCX)

S2 Table Parasite load in blood samples, grouped by *T*. *cruzi* Discrete Typing Units. (DOCX)
