# Supplementary material for: Exploring the parasite load and molecular diversity of Trypanosoma cruzi in patients with chronic Chagas disease from different regions of Brazil
Source: PLoS Negl Trop Dis. 2018 Nov 12;12(11):e0006939. doi: 10.1371/journal.pntd.0006939 (PMC6258420; doi:10.1371/journal.pntd.0006939)
Supplement: S1 Table — (DOCX) [file pntd.0006939.s001.docx]

**Table S1. DNA sequences identification from PCR products for SL-IR I and II, 24Sα and A10 targets.**

| **Strains** | **DTU** | **NCBI access number** | **Description** | **Query cover** | **Identity** | **E- value** |
| --- | --- | --- | --- | --- | --- | --- |
| **Intergenic region of spliced leader gene (SL-IR I and II)** | | | | | | |
| **Dm28c** | I | EF626693.1 | Trypanosoma cruzi trans-spliced leader sequence mini-exon repeat region | 100% | 100% | 4e-13 |
| **Y** | II | U57984.1 | Trypanosoma cruzi strain CL spliced leader gene repeat sequence | 100% | 95% | 1e-100 |
| **24Sα ribosomal RNA gene** | | | | | | |
| **Y** | II | GQ303145.1 | Trypanosoma cruzi 24S alpha ribosomal RNA gene, partial sequence | 97% | 95% | 3e-54 |
| **3663** | III | GQ303145.1 | Trypanosoma cruzi 24S alpha ribosomal RNA gene, partial sequence | 98% | 93% | 4e-85 |
| **Bug2149** | V | GQ303145.1 | Trypanosoma cruzi 24S alpha ribosomal RNA gene, partial sequence | 100% | 96% | 1e-91 |
| **CL** | VI | GQ303145.1 | Trypanosoma cruzi 24S alpha ribosomal RNA gene, partial sequence | 100% | 88% | 1e-21 |
| **A10 nuclear fragment** | | | | | | |
| **Y** | II | \| AJ133198.1 \|  \|  \| \| --- \| --- \| --- \| | Trypanosoma cruzi (CL Brener) RAPD fragment A10e | 100% | 99% | 3e-26 |
| **CL** | VI | EF394303.1 | Trypanosoma cruzi isolate PAH265 RAPD marker A10 fragment | 100% | 99% | 1e-38 |
