# Supplementary material for: Exploring the parasite load and molecular diversity of Trypanosoma cruzi in patients with chronic Chagas disease from different regions of Brazil
Source: PLoS Negl Trop Dis. 2018 Nov 12;12(11):e0006939. doi: 10.1371/journal.pntd.0006939 (PMC6258420; doi:10.1371/journal.pntd.0006939)
Supplement: S2 Table — (DOCX) [file pntd.0006939.s002.docx]

**Table S2. Parasite load in blood samples, grouped by *T. cruzi* Discrete Typing Units.**

| **Patient**  **#** | ***T. cruzi* DTU** | **Parasite load mean**  **(Par. Eq./ mL)** | **Standard**  **deviation** | **Parasite load range**  **[Minimum – Maximal]** |
| --- | --- | --- | --- | --- |
| **1** | II | 13.07 | 2.29 | [2.90 – 15.82] |
| **2** | II | 2.90 | 1.15 |  |
| **3** | II | 4.45 | 1.86 |  |
| **4** | II | 15.82 | 2.08 |  |
| **5** | II | 5.44 | 6.75 |  |
| **6** | II | 9.69 | 1.37 |  |
| **7** | II/V/VI | 0.12 | 0.16 | [0.12 – 1.59] |
| **8** | II/V/VI | 1.09 | 0.42 |  |
| **9** | II/V/VI | 1.59 | 0.50 |  |
| **10** | II/VI | 1.39 | 0.96 | [1.39 – 25.32] |
| **11** | II/VI | 20.05 | 2.75 |  |
| **12** | II/VI | 4.44 | 2.66 |  |
| **13** | II/VI | 25.32 | 3.50 |  |
| **14** | II+VI | 0.95 | 0.08 | [0.30 – 16.52] |
| **15** | II+VI | 14.86 | 3.36 |  |
| **16** | II+VI | 1.82 | 2.57 |  |
| **17** | II+VI | 1.48 | 1.49 |  |
| **18** | II+VI | 16.52 | 7.78 |  |
| **19** | III+VI | 0.30 | 0.37 |  |
| **20** | V | 3.12 | 3.10 | [1.55 – 3.12] |
| **21** | V | 1.55 | 1.63 |  |
| **22** | VI | 0.14 | 0.20 | [0.14 – 153.66] |
| **23** | VI | 2.37 | 3.35 |  |
| **24** | VI | 2.54 | 3.59 |  |
| **25** | VI | 153.66 | 27.49 |  |
| **26** | VI | 0.57 | 0.81 |  |
| **27** | VI | 2.27 | 2.35 |  |
| **28** | VI | 2.59 | 3.44 |  |
| **29** | VI | 16.68 | 3.64 |  |
| **30** | VI | 1.27 | 1.79 |  |
| **31** | VI | 12.58 | 1.78 |  |
| **32** | VI | 2.32 | 0.41 |  |
| **33** | VI | 2.34 | 0.93 |  |
| **34** | VI | 5.18 | 7.32 |  |
| **35** | VI | 0.69 | 0.97 |  |
